# Prognosis Signature of Cuprotosis-Related lncRNAs Associated with Kidney Renal Clear Cell Carcinoma

**DOI:** 10.1155/2022/6004852

**Published:** 2022-11-17

**Authors:** Yiren Gao, Zhen Jia

**Affiliations:** ^1^Traditional Chinese Medicine Department, The Affiliated Hospital to Changchun University of Chinese Medicine, Changchun, Jilin, China; ^2^Department of Peripheral Vascular Diseases, First Affiliated Hospital, Heilongjiang University of Traditional Chinese Medicine, Harbin, China

## Abstract

Cuprotosis is a novel cell death mechanism that can be explored to treat various tumors. A few studies on the role of cuprotosis-related long noncoding RNA (lncRNA) in the development and prognosis of kidney renal clear cell carcinoma (KIRC) have been reported. We aimed to study the relationship between the prognosis of patients suffering from KIRC and lncRNAs associated with cuprotosis. The Cancer Genome Atlas (TCGA) database was analyzed, and the transcriptome data and clinical information on the patients with KIRC were obtained. The cuprotosis-related lncRNAs were identified by using Pearson correlation analysis, and the significant changes in the lncRNAs associated with KIRC were studied by conducting the *T*-test. The cuprotosis-related lncRNAs with KIRC prognostic values were identified by using the univariate Cox analysis, least absolute shrinkage and selection operator (LASSO), and support vector machine (SVM) methods. A prognostic marker composed of three cuprotosis-related lncRNAs was identified following the multivariate regression analysis method. Patients with KIRC were divided into two groups based on the expression characteristics of three cuprotosis-related lncRNAs by using the *K* nearest neighbor (KNN) cluster analysis method. Significant differences in survival were observed between the two groups. In addition, the results obtained following the independent prognostic analysis of the risk score (RS) and clinical correlation revealed that the three cuprotosis-related lncRNA prognostic markers could accurately predict the prognosis of patients with KIRC. The results reported herein provide new insights into the pathogenesis of KIRC and the contribution of lncRNAs associated with cuprotosis. The results also helped identify a prognostic indicator that could potentially provide information for KIRC treatment.

## 1. Introduction

Kidney renal clear cell carcinoma (KIRC) accounts for 80–86% of cancer cases associated with the urinary system. This is one of the most prevalent forms of urinary system-related cancers in the world [1, 2]. KIRC patients are not sensitive enough to chemotherapy, radiotherapy, and target therapy [3]. The lack of accurate molecular targets results in a low survival rate for advanced-stage KIRC patients [4]. Therefore, it is important to identify effective biomarkers to accurately predict the prognosis of patients suffering from KIRC.

Copper is an important element that influences the survival and growth of an organism. It significantly affects various life activities. Under normal physiological conditions, a low concentration of copper ions is present in the organisms under dynamic conditions. The abnormal accumulation of copper ions causes copper toxicity that eventually induces cell death [5]. It has been previously reported that the level of copper in normal patients is significantly lower than the level of copper in patients with tumors [6, 7]. Cuprotosis is a novel mechanism that causes cell death. This mechanism can be analyzed to develop new methods for the prevention and treatment of tumors [8, 9]. It has been previously reported that cuprotosis regulatory genes significantly affect the prognosis of KIRC patients [10]. Cuprotosis should be explored further to develop new strategies for the treatment of KIRC.

LncRNA is a type of RNA that is more than 200 nts long and does not encode proteins [11]. It accounts for a large proportion of RNAs in the human transcriptome. It has been reported in recent years that lncRNAs participate in various important regulatory processes such as transcriptional activation, genome imprinting, and chromatin modification [12]. It has also been reported that lncRNAs can regulate gene expression at the post-transcriptional, transcriptional, and epigenetic levels. Gene expression and the process of development are controlled by a complex and precise regulatory mechanism associated with the lncRNAs that participate in numerous biological processes such as metastasis, apoptosis, invasion, and tumor proliferation [13]. It has been widely reported that lncRNAs related to different cell death modes can accurately predict the prognosis of patients with tumors. For example, linc01871 and sema3b-as1, which are associated with autophagy, can effectively predict the survival of breast cancer patients [14, 15]. Accurate prediction of the survival rate of patients suffering from lung cancer can be realized by analyzing the ferroptosis-related ac026355.1, al606489.1, linc02081, ac106047.1, and ac090559.1 [16]. Coke death-related linc0900, CRNDE, and lbx2-AS1 are closely associated with the prognosis of glioma [17, 18]. However, the relationship between cuprotosis-related lncRNA and tumors needs to be studied further.

The lncRNAs associated with cuprotosis and renal clear cell carcinoma were systematically identified, and the prognosis signature of cuprotosis-related lncRNA was constructed by analyzing the clinical characteristics of KIRC patients in TCGA database. The ability of lncRNAs to accurately and independently predict the prognosis of KIRC patients was also evaluated. The results reported herein helped improve the effectiveness of the individualized treatment methods and the prognosis of patients [19].

## 2. Materials and Methods

### 2.1. Data Collection

Data corresponding to the transcriptome expression profile of the patients suffering from KIRC were downloaded from TCGA database. Data on 530 KIRC patient tissue samples, 72 normal tissue samples, and their supporting clinical data were downloaded. The annotation information of lncRNA was downloaded from the Genecode database [20].

### 2.2. Identification of Differential Cuprotosis-Related lncRNA in KIRC

We obtained cuprotosis-related genes from Peter's study [21] and got the gene expression and the lncRNA expression characteristics of the KIRC patients. We used the Pearson correlation analysis method to calculate the correlation between the expression value of cuprotosis-related genes and lncRNAs associated with KIRC (*r* > 0.5, *p* < 0.05). Following this, the *T*-test, fold-change test, and Benjamin–Hochberg multiple tests were conducted to correct and identify the differential lncRNAs associated with KIRC (false discovery rate (FDR) < 0.05; |log^2^ (FC) |>1).

### 2.3. Identification and Construction of Prognostic Markers for KIRC

Univariate Cox regression models (survival and surviviner packages; *R* language) were used to analyze differential lncRNAs to identify lncRNAs associated with the survival of cancer samples (*p* < 0.05). Risk-related lncRNAs and protective lncRNAs (HR < 1) were identified based on the hazard ratios (HR; >1 for risk-related lncRNAs; <1 for protective lncRNAs). The identified lncRNA was analyzed by using the LASSO model (R language; glmnet package) and SVM–RFE to identify the prognostic markers of KIRC [22–24]. The Lasso model was constructed. The best *λ* value was selected based on the analytical results and results obtained from comparison tests to identify the characteristic lncRNA in the Lasso model. During the process of SVM–RFE analysis, lncRNA was finally obtained as the feature of SVM-RFE by comparing the correct rate and error rate under conditions of different feature numbers. The intersection of the results obtained by using the lasso model and SVM-RFE was considered to obtain the candidate KIRC-related lncRNA. Subsequently, the multivariable Cox regression analysis (survival and surviviner language packages; *R*) method was used to analyze the candidate lncRNAs associated with KIRC to evaluate their contribution as integrated prognostic factors toward the survival of the patients. lncRNAs with *p* < 0.05 and HR < 1/HR > 1 were selected as the lncRNAs associated with the prognosis of KIRC. The effect of candidate lncRNAs on the prognosis of KIRC was analyzed by analyzing the Kaplan–Meier (survival and surviviner language packages; *R*) survival curve.

### 2.4. Evaluation of the Effect of Prognostic lncRNA

The KNN clustering algorithm (CV.kNN function; *R* language class package; parameter: *k* = 2) was used for analysis, the expression values of the candidate lncRNAs were considered as the clustering feature, and the samples obtained from the KIRC cancer patients were clustered into two categories. Based on the lncRNA expression of each cluster subgroup, the cluster subgroups were divided into the high-expression and low-expression groups. The overall survival (OS) of the patients belonging to the low-expression and high-expression groups was compared by analyzing the Kaplan–Meier (*R* language; survival and surviviner language packages) survival curve and conducting the bilateral time series tests. The *R* language (RTsne function in the RTsne *R* package; *R* language) was used to execute the t-Distributed Stochastic Neighbor Embedding (t-SNE) technique for the two-dimensional reduction processing of the expression profile data of KIRC prognosis-related lncRNA. The visual KNN method was used for clustering. Subsequently, the multivariate Cox regression analysis (survival and surviviner language packages; *R* language) method was used to analyze the KIRC prognosis-related lncRNAs to understand their contribution as integrated prognostic factors in determining the survival of patients. Based on the results obtained by using the multivariate Cox regression model (survival and surviviner language packages; *R* language), the survival status and prognosis time of each KIRC patient affected by KIRC prognosis-related lncRNA were predicted, and the effect of the prognosis model was evaluated by analyzing the receiver operating characteristic curve (ROC).

### 2.5. Construction of the Forecast Nomogram

RS was established based on the KIRC prognosis-related lncRNAs signature. Patients were divided into low-risk and high-risk groups using the value of the median RS as the threshold. The Kaplan–Meier curve was analyzed to analyze the survival difference between patients belonging to the low-risk and high-risk groups. The 1-year, 3-year, and 5-year OS was predicted by constructing a nomogram. RS was integrated [25], and other clinicopathological factors such as the *T* stage, Grade grading, and Stage staging parameters were analyzed to arrive at the results. In addition, the calibration and ROC curves were analyzed to determine the performance of the model.

## 3. Results

### 3.1. Identification of Differentially Cuprotosis-Related lncRNAs Associated with KIRC

**Figure 1:**
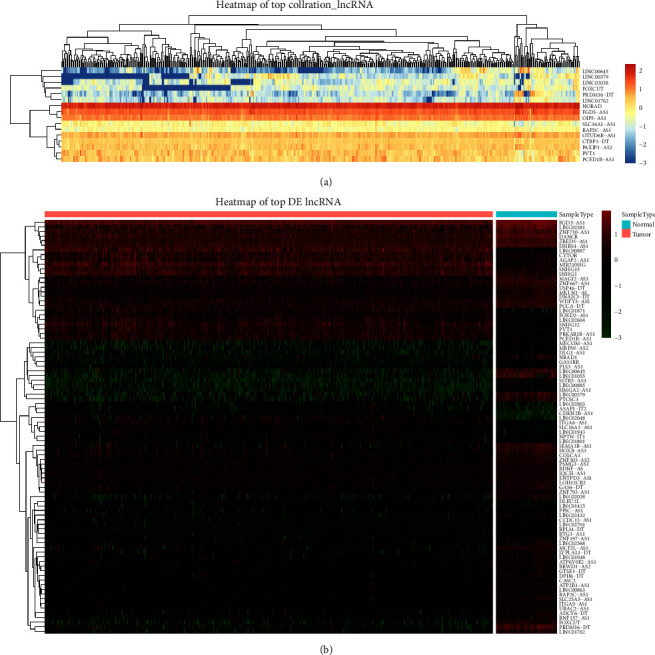
Select cuprotosis-related lncRNAs and identify differentially expressed cuprotosis-related lncRNAs in KIRC. (a) Heat map of top cuprotosis-related lncRNAs associated with KIRC. (b) Heat map presenting the differential expression of the cuprotosis-related lncRNAs associated with KIRC.

First, 602 sample data for KIRC were obtained from TCGA database. The data corresponding to 530 patients and 72 normal subjects were obtained to conduct the studies. After integrating the cuprotosis-related genes (CDKN2A, fdx1, DLD, DLAT, lias, GLS, lipt1, MTF1, PDHA1, and PDHB), we identified 213 cuprotosis-related lncRNAs associated with KIRC using the Pearson correlation analysis (*p* < 0.05, *r* > 0.5) method. The results are presented in Figure 1(a). Following this, the differential expression of cuprotosis-related lncRNAs was analyzed by conducting the *T*-test. The results are presented in Figure 1(b). Subsequently, 89 differentially expressed lncRNAs (*p* < 0.05 and FC > 1/FC < -1 lncRNAs) were obtained.

## 4. Identification of Cuprotosis-Related lncRNAs with Prognostic Value for KIRC

**Figure 2:**
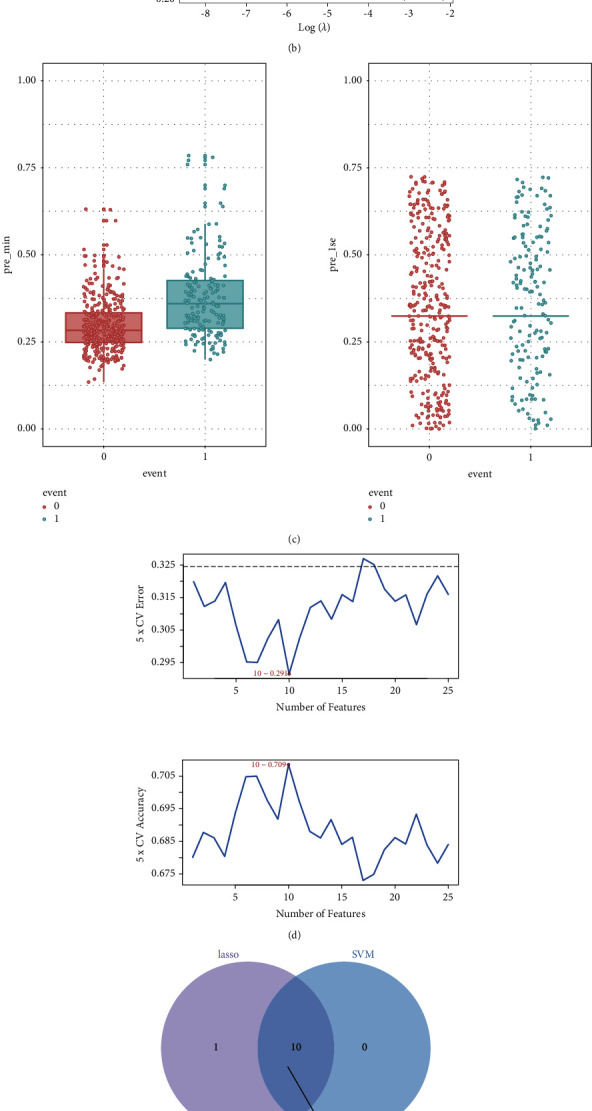
LncRNA for the determination of the prognostic value in KIRC. (a) Process of model parameter selection during the use of the Lasso method. (b) Dose K-fold cross-validation for the Lasso model to select model parameters. (c) Comparison graph corresponding to the 1se model and min model in Lasso. (d) Feature graph of the optimal solution for feature selection during the use of the SVM algorithm. (e) Venn graph representing gene intersection between the Lasso model and the SVM algorithm.

The univariate Cox regression analysis method was used to analyze the differential cuprotosis-related lncRNAs associated with KIRC to identify the KIRC prognosis-related lncRNAs and the cuprotosis-related lncRNAs. LncRNAs characterized by *p* < 0.05 and HR > 1/HR < 1 were selected, and finally, 30 lncRNAs were obtained. To identify the valuable prognostic markers, the Lasso and SVM methods were used to construct models and analyze the potential role of 30 lncRNAs. The results are presented in Figures 2(a)–2(c). The Lasso analysis (the coefficient obtained by using the Lasso analysis method was 11) method was used for data analysis, and the results of the ls1 and min models were compared. The results obtained by using the min model were selected to obtain 11 lncRNAs. The results obtained by using the SVM feature selection analysis method (feature selection 10) are presented in Figure 2(d), and finally, 10 lncRNAs were identified. Following the intersection of the two model genes, 10 lncRNAs (Figure 2(e)), namely, LINC01871, LINC01943, RPL34-DT, PRKAR1B-AS1, PSMG3-AS1, SNHG15, LINC02604, AGAP2-AS1, LINC01801, and RAP2C-AS1, were finally obtained, which were considered to be the most effective among the cuprotosis-related lncRNAs associated with the prognosis of KIRC.

### 4.1. Evaluation of the lncRNAs Related to Cuprotosis Signature

**Figure 3:**
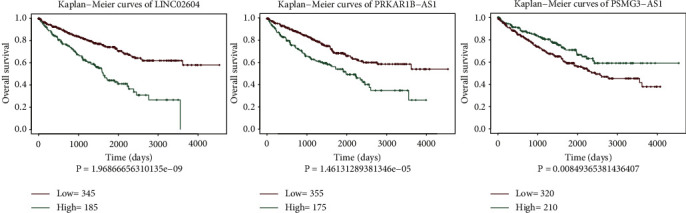
Identification of the KIRC prognosis signature. (a) Kaplan–Meier survival analysis chart of significant lncRNAs.

**Figure 4:**
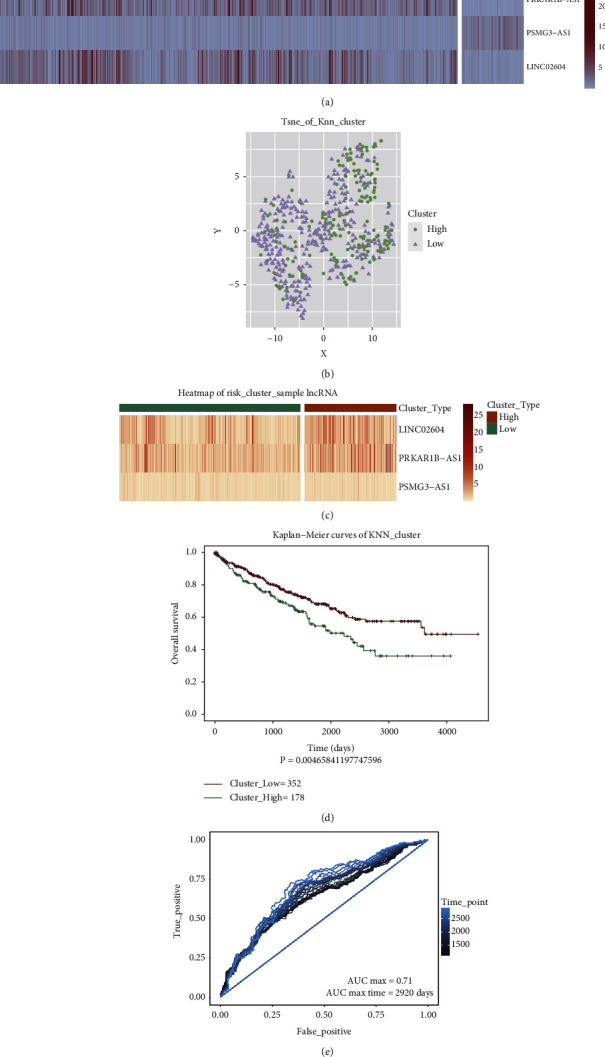
Construction and evaluation of the prognostic signatures. (a) Heat map of signatures. (b) KNN clustering-based scatter diagram. (c) Clustering analysis of signatures. (d) Survival analysis of the two types of samples after clustering. (e) Analysis of the classification efficiency for signatures.

The 10 lncRNAs that were identified were analyzed by using the multivariate Cox regression analysis methods. Three cuprotosis-related lncRNAs, namely, psmg3-as1, linc02604, and prkar1b-as1 that could be used as KIRC prognostic markers, were identified. Among them, linc02604 and prkar1b-as1 were considered to be adverse prognostic factors (HR > 1, Table 1), and psmg3-as1 was considered to be a favorable prognostic factor (HR < 1, Table 1). Results obtained by analyzing the Kaplan–Meier curve (Figure 3(a)) revealed that the three cuprotosis-related lncRNAs correlated significantly with the prognosis of the patients suffering from KIRC. Subsequently, three lncRNAs related to cuprotosis were established as independent predictors of KIRC patients (Figure 4(a)). The expression levels of the three lncRNAs related to cuprotosis associated with KIRC were significantly different from each other. The KNN method was used as the clustering method to cluster patients suffering from KIRC. The results are presented in Figures 4(b) and 4(c). The method could be used to effectively divide KIRC patients into two categories. The results obtained by analyzing the Kaplan–Meier curve revealed that the survival rate of the two categories of KIRC patients was significantly different from each other (Figure 4(d)). The ROC curves were evaluated (area under curve (AUC) = 0.71), and the results revealed that the classification efficiency was good (Figure 4(e)).

### 4.2. Independent Prognostic Analysis for RS and the Clinical Relevance

psmg3-as1, linc02604, and prkar1b-as1 were used to construct a prognosis model to develop a clinically feasible method that could be used for predicting the survival probability of patients. The RS of each patient was calculated based on the Cox coefficient and expression level of the prognosis model gene. The RS formula can be expressed as follows:

0.3939 × PRKAR1B-AS1 + (−0.69962 × PSMG3-AS1) + (0.39786 × LINC02604).

**Figure 5:**
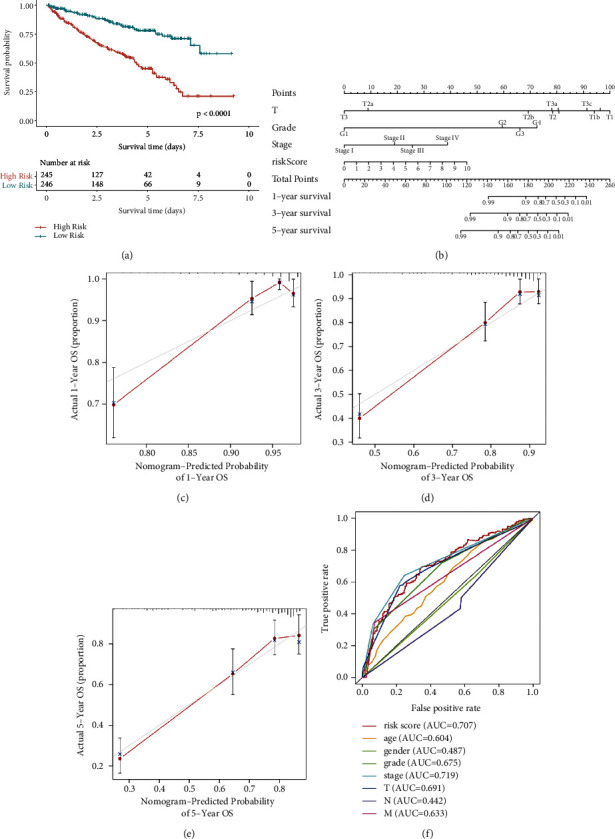
Analysis of the prognosis model. (a) Kaplan–Meier curves corresponding to the OS for high-risk and low-risk groups stratified based on RS (*p* < 0.0001). (b) A clinical prognostic nomogram was developed to predict 1 -year, 3 -year, and 5-year survival. Calibration curves showing nomographic predictions for 1-year (c), 3-year (d), and 5-year (e) survival. (f) Prediction of the 5-year OS based on the RS, age, sex, grade, stage, and TNM stage.

The patients were divided into low-risk (*n* = 246) and high-risk groups (*n* = 245) using the median RS value as the threshold. Analysis of the Kaplan–Meier curve revealed a significant difference between the survival rates of the low-risk and the high-risk groups for KIRC patients (Figure 5(a)). Combined with other clinical features, a prediction model was constructed by constructing a nomogram (Figure 5(b)). Analysis of the 1 -year, 3 -year, and 5-year nomogram calibration curves (Figures 5(c)–5(e)) revealed that the estimated survival rate became closer to the actual survival rate with the progress of time. The ROC curve for 5-year survival was also generated (Figure 5(f)). The AUC value of RS was significantly higher than that recorded for most clinical features, proving that the prognosis model could accurately predict the KIRC survival rate.

## 5. Discussion

KIRC is a common subtype of renal cell carcinoma that is characterized by a high incidence rate and mortality [26, 27]. It is important to develop effective prognostic models for KIRC. Cuprotosis is a new novel copper ion-dependent cell death type being regulated in tumor cells, and this is different from the common cell patterns such as apoptosis, pyroptosis, necroptosis, and ferroptosis. The process of cuprotosis is closely related to mitochondrial respiration. The copper that is too abundant within cells can be transported to the mitochondria by ionophores and directly bind to lipoylated components of the tricarboxylic acid cycle, resulting in the accumulation of lipoylated proteins and loss of iron–sulfur cluster proteins, which leads to proteotoxic stress and ultimately to cell death. Interestingly, like with death patterns, cuprotosis-related genes have been reported to play a key role in the processes associated with tumor regulation, such as KIRC. It has been previously reported that cuprotosis-related genes can predict the survival of patients suffering from KIRC [10]. Therefore, cuprotosis-related genes are potential therapeutic targets for KIRC. However, to the best of our knowledge, few researchers have explored the correlation between cuprotosis and lncRNAs. We used the bioinformatics analysis method to systematically analyze the role of cuprotosis-related lncRNAs in the field of prognosis of KIRC. In addition, the cuprotosis-related lncRNAs scoring system was proposed to evaluate individual cuprotosis-related lncRNAs to improve understanding of the prognosis of KIRC.

To the best of our knowledge, few researchers have explored the correlation between cuprotosis and lncRNAs. We observed that a certain number of cuprotosis-related lncRNAs were associated with the changes in KIRC, and these were related to the prognosis of KIRC patients. This also suggested that cuprotosis-related molecules affected the development and prognosis of KIRC. Firstly, the cuprotosis-related lncRNAs differentially expressed in KIRC were identified, following which 10 cuprotosis-related lncRNAs with prognostic values in KIRC were obtained following the Cox univariate analysis Lasso and SVM methods. Subsequently, psmg3-as1, linc02604, and prkar1b-as1 were selected as the prognostic signatures based on the results obtained by using the multivariate Cox regression analysis method. We used the prognostic signatures to divide KIRC patients into two groups following the KNN clustering method. It was observed that the prognostic cuprotosis-related lncRNAs constructed by using psmg3-as1, linc02604, and prkar1b-as1 could significantly affect the survival of the two groups. What is more, in a reported study, the marker gene FDX1 associated with cuprotosis which has been discovered can influence the proliferation of KIRC cells. In our study, prognostic cuprotosis-related lncRNAs were discovered, which can target the key genes of cuprotosis.

A nomogram can function as a reliable and effective clinical tool that can be used to predict the survival time of tumor patients. Therefore, a robust nomogram health map consisting of multiple clinical variables was developed. Analysis of the calibration map revealed that the actual 1 -year, 3 -year, and 5-year survival rates were comparable to the predicted survival rates. Among the three potential prognostic markers that were identified, LINC02604 was found to be an effective marker for the prognosis of colon cancer [28], and psmg3-as1 was found to play a key regulatory role in the occurrence and development of various tumors, such as lung cancer [29,30], breast cancer [31], and ovarian cancer [32]. The results helped us study their role in the development and prognosis of KIRC. In conclusion, it can be inferred that the lncRNA prognostic markers associated with cuprotosis could accurately predict the survival of KIRC patients. It was observed that the markers exhibited great potential for clinical application and could be used to realize individualized prognosis and treatment.

There are several limitations to this study. First, the results reported herein should be further validated using other sets of independent data to determine the robustness of the cuprotosis-related lncRNA prognostic factors. Secondly, further biochemical experiments should be conducted by using various techniques such as immunohistochemistry, flow cytometry, and real-time fluorescent quantitative polymerase chain reaction (PCR). The method of clinical data analysis should also be conducted to validate the reported results.

## 6. Conclusion

It can be concluded that the prognostic characteristics of the cuprotosis-related lncRNAs associated with KIRC were systematically analyzed. The results revealed that cuprotosis-related lncRNA prognostic markers could accurately predict the prognosis and survival of KIRC patients. The results also revealed that the three lncRNAs related to cuprotosis were promising targets for KIRC treatment.

## Figures and Tables

**Table 1:** Multivariate Cox regression analysis.

	*p* value	HR
AGAP2-AS1	0.041	1 (1–1.1)
LINC02604	0.0034	1.1 (1–1.2)
RPL34-DT	0.21	0.081 (0.0016–4.1)
PRKAR1B-AS1	0.00067	1.1 (1–1.1)
PSMG3-AS1	0.028	0.64 (0.43–0.95)
LINC01943	0.71	1 (0.83–1.3)
RAP2C-AS1	0.63	1.2 (0.54–2.8)
LINC01801	0.16	0.75 (0.5–1.1)
LINC01871	0.57	1 (0.94–1.1)
SNHG15	0.37	1 (0.98–1.1)

## Data Availability

Previously reported (high-throughput sequencing) data used to support this study were down from TCGA (https://cancergenome.nih.gov/) database. These prior studies (and datasets) are cited at relevant places within the text as references [Long Non-Coding RNA Profile Study Identifies an Immune-Related lncRNA Prognostic Signature for Kidney Renal Clear Cell Carcinoma. Front Oncol. 2020 Aug 20;10 : 1430. doi: 10.3389/fonc.2020.01430. PMID: 32974157.].
